# Impact of fluid management during the three ICU days after admission in patients with ARDS

**DOI:** 10.1186/cc14028

**Published:** 2014-12-03

**Authors:** A Murai, H Ishikura, N Matsumoto, Y Nakamura, D Ohta, K Muranishi, Y Izutani, T Nishida

**Affiliations:** 1 Department of Emergency and Critical Care Medicine, Faculty of Medicine, Fukuoka University, Fukuoka, Japan

## Introduction

The optimal evaluation of fluid resuscitation in patients with acute respiratory distress syndrome (ARDS) remains to be clearly elucidated. The purpose of this study was to clarify the influence of fluid overload in patients with ARDS.

## Methods

Two hundred and seven ARDS patients admitted to the ICUs of 23 tertiary referral hospitals in Japan were enrolled in this study. These patients were divided into survivor (survival group, *n *= 137) or nonsurvivor (nonsurvival group, *n *= 70) groups according to the 28-day mortality. All patients received mechanical ventilation and also underwent transpulmonary thermodilution monitoring. The data for analysis were collected for three consecutive days from time of admission.

## Results

On the second hospital day, the extravascular lung water index of the nonsurvival group was significantly higher than that of the survival group (18.6 ± 9.4 ml/kg vs. 15.4 ± 6.3 ml/kg, *P *= 0.03). Moreover, regarding the first 3-day cumulative fluid balance, the nonsurvival group was significantly higher than survival group (5.1 ± 4.3 l vs. 3.5 ± 0.4 l, *P *= 0.015). We suspected that these results might be related to the cardiovascular and/or renal function. We therefore excluded any patients with a score of three or more regarding the cardiovascular and/or renal criteria about the Sequential Organ Failure Assessment score. Thereafter, we confirmed the results to be similar for the first 3-day cumulative fluid balance between the two groups (3.8 ± 1.6 l vs. 2.2 ± 4.0 l, *P *= 0.0339). A stepwise logistic regression analysis identified the 3-day cumulative fluid balance to be an independent risk factor for the 28-day mortality (adjusted odds ratio: 1.0001, 95% CI: 1.000017 to 1.000217, *P *= 0.0252).

## Conclusion

Our findings suggest that excessive fluid resuscitation may therefore have a negative impact on the 28-day mortality for patients with ARDS.

